# Place of Delivery Associated With Postnatal Care Utilization Among Childbearing Women in Zambia

**DOI:** 10.3389/fpubh.2018.00094

**Published:** 2018-04-06

**Authors:** Charles Chungu, Mpundu Makasa, Mumbi Chola, Choolwe Nkwemu Jacobs

**Affiliations:** ^1^Ministry of Health, Muchinga Provincial Health Office, Chinsali, Zambia; ^2^Department of Epidemiology and Biostatistics, School of Public Health, University of Zambia, Lusaka, Zambia; ^3^Department of Community and Family Medicine, School of Public Health, University of Zambia, Lusaka, Zambia

**Keywords:** postnatal care, place, delivery, Zambia, utilization, rural women

## Abstract

**Objective:**

Postnatal care (PNC) utilization is critical to the prevention of maternal morbidity and mortality. Despite its importance, the proportion of women utilizing this service is still low in Zambia. We investigated if place of delivery was associated with PNC utilization in the first 48 h among childbearing women in Zambia.

**Methods:**

Data from the 2013/14 Zambia Demographic and Health Survey for women, aged 15–49 years, who reported giving birth in the 2 years preceding the survey was used. The data comprised of sociodemographic and other obstetric data, which were cleaned, recoded, and analyzed using STATA version 13 (Stata Corporation, College Station, TX, USA). Multivariate logistic regression was used to examine the association of place of delivery and other background variables.

**Results:**

Women who delivered in a health facility were more likely to utilize PNC in the first 48 h compared to those who did not deliver in a health facility: government hospital (AOR 7.24, 95% CI 4.92–11.84), government health center/clinic (AOR 7.15 95% CI 4.79–10.66), other public sector (AOR 23.2 95% CI 3.69–145.91), private hospital/clinic (AOR 10.08 95% CI 3.35–30.35), and Mission hospital/clinic (AOR 8.56 95% CI 4.71–15.53). Additionally, women who were attended to by a skilled personnel during delivery of the baby were more likely to utilize PNC (AOR 2.30, 95% CI 1.57–3.37). Women from rural areas were less likely to utilize PNC in the first 48 h (AOR 0.70, 95% CI 0.53–0.90).

**Conclusion:**

Place of delivery was found to be linked with PNC utilization in this population although access to health care is still driven by inequity-related dynamics and imbalances. Given that inequity stresses are heaviest in the rural and poor groups, interventions should aim to reach this group.

**Significance:**

The study results will help program managers to increase access to health facility delivery and direct interventional efforts toward the affected subpopulations, such as the young and rural women. Furthermore, results will help promote maternal health education on importance of health facility delivery and advise policy makers and program implementers.

## Introduction

The World Health Organization reports that of the 289,000 maternal deaths that occur each year, worldwide (1), 50 to 71% occur within the postnatal period (2). On the other hand, 2.9 million neonatal deaths occur in the first week of life on an annual basis globally (3). Unfortunately, 99% of these maternal and neonatal deaths occur in low- and middle-income countries (4) to which Zambia belongs. According to the 2013–2014 Zambia Demographic and Health Survey (ZDHS), Zambia had a maternal mortality rate of 398 maternal deaths per 100,000 live births (5).

Postnatal care (PNC) is critical to the survival of both the mother and her newborn baby. PNC is the service provided to both the mother and her newborn baby within the first 6 weeks of a child’s birth (1). The service includes; an integrated package of routine maternal and neonatal care as well as extra care for those mothers and neonates who may be considered particularly vulnerable. The interventions include the umbilical cord care; special care for preterm, low-birth-weight and HIV-infected neonates (6), screening and treatment of infections and postnatal growth restriction, as well as assessment for factors predisposing to infant anemia (7). In addition, health education is provided to mothers on danger signs for her neonate, such as convulsions or problems with feeding (1) and to seek prompt treatment. In Zambia, PNC is scheduled as recommended by the Ministry of Health and as cited by Central Statistical Office (2014) into; within the first 6 h after birth, within 48 h, after 6 days, and then finally within the last 6 weeks after the birth of the baby. The importance of PNC services cannot be overemphasized; however, utilization coverage remains poor in developing countries (8). For example, in a recently conducted review of Demographic and Health Surveys from 13 African countries, of those who deliver at home only 13% received PNC services (9). In Zambia, maternal PNC coverage within the first 2 days has been estimated to be at 63%, which is below the national minimum target of 80% (4, 10).

Some studies have shown that place of delivery is associated with maternal PNC utilization in the first 48 h (2, 11). Women who delivered in health facilities were more likely to utilize PNC than those who did not. In Zambia, place of delivery was found to predict PNC utilization in the first 48 h after delivery of the baby in four rural districts (12). The study only considered four rural districts which were not representative of the countries rural and urban areas, however, the ZDHS on which this study is based, covered the entire country. This study aimed to determine if place of delivery is associated with maternal PNC service utilization within the first 48 h after birth in Zambia. The study involved using the nationally representative 2013–2014 ZDHS data. Understanding the association between place of delivery and maternal PNC utilization in the first 48 h after birth in Zambia would be important in efforts to improve coverage and utilization of maternal PNC services, and consequently reduction of maternal deaths.

## Methodology

### The 2013/14 ZDHS Design

This was a cross-sectional study based on the 2013/14 ZDHS. The 2013/14 ZDHS is a nationally representative survey of 16,411 women aged 15–49 years and 14,773 men aged 15–59 years. The population that was focused on was that of females of the reproductive age, between 15 and 49 years. This study included all women who had a child within the 2 years preceding the survey. The utilization of PNC was considered for the last birth prior to the survey. All women age 15–49 that were either permanent residents of the households in the sample or visitors present in the household on the night before the survey were eligible to be interviewed. All women who had a child within 2 years preceding the survey but did not attend PNC were excluded. The ZDHS used a two-stage stratified sampling. For the selection of clusters and households, probability proportional to size at first stage, and equal probability systematic sampling was applied at second stage. The details of the ZDHS methodology are recorded in the reports (5).

### PNC Utilization Design

The PNC utilization study was based on data that was extracted from the 2013/14 ZDHS Women’s questionnaire. Women who reported having given birth two years prior to the survey and utilized PNC defined the sample of this study (*n* = 5,074). From these women aged 15–49 years who were captured in the survey, the proportion that attended PNC in the first 48 h after birth of the baby comprised *de facto* eligible sample. The explanatory variables included; mother’s age at birth (in years, ordered), birth order, place of delivery (health facility delivery or other place, e.g., home), residence (urban or rural), maternal education and wealth status, maternal and paternal occupation, birth attendance during delivery (skilled attendance), ANC timing, marital status, distance to a health facility, and being told about pregnancy complications. These factors were found to be significantly associated with PNC utilization in studies done for example, in Nepal and Tanzania (13, 14). The outcome of interest was PNC utilization (either in the first 48 h or after 48 h following the delivery of the baby) by women aged 15–49 years who had a baby 2 years prior to the survey considering the most recent birth.

### Statistical Analysis

Descriptive and inferential statistics were used to examine if place of delivery was associated with PNC utilization in the first 48 h after birth. In the first step, univariate analysis (initially by cross tabulations by Pearson’s chi-squared test) and later multiple logistic regression, incorporating survey weights were performed to examine if place of delivery was associated with PNC in the first 48 h after birth. A *p* value of <0.05 was considered significant with 95% confidence interval (95% CI). STATA version 13 (Stata Corporation, College Station, TX, USA) was used for all analyses in this study.

### Ethics

Ethical approval for the 2013/14 ZDHS was obtained from the Tropical Diseases Research Centre in Ndola, Zambia and the US Centre for Disease Control and Prevention Atlanta Research Ethics Review Board. Participation in the survey was based on informed and voluntary consent. The re-analysis of the data reported in this study did not infringe on participants’ privacy and was judged to pose no risk, since these data were already de-identified, approved, and made available for public use. Additionally, clearance was obtained from Excellency in Research Ethics and Science Committee that granted approval to conduct this study on the factors associated with maternal PNC utilization based on the 2013/14 ZDHS (Ref. no. 2016-June-014).

## Results

### Characteristics of Participants

A total of 5,074 women were considered in this study. The least (13%) proportion was represented by those between 15 and 19 years, while those between 20 and 24 years represented a quarter of the total sample. Only 22% were first time mothers and 80% were married. Most (88%) of the mothers were told about pregnancy complications during their visit for antenatal care. Nearly 28% of the women did not deliver in health a facility. The majority (47%) delivery in government health centers or health post. About 69% were attended to by a skilled personnel. Table 1 presents the characteristics of the respondents.

**Table 1:** Basic characteristics of childbearing women utilizing postnatal care (PNC) in Zambia.

Characteristic	Weighted % (n*)	95% CI
Age (in years, grouped)		
15–19	13.3 (675)	(12.4–14.6)
20–24	25.8 (1,307)	(24.6–27.0)
25–29	24.8 (1,257)	(23.6–26.0)
30–34	18.5 (937)	(17.4–19.6)
35–49	17.7 (898)	(16.7–18.8)
PNC timing		
Within 48 h	63.4 (3,220)	(61.9–64.6)
After 48 h	36.6 (1,854)	(35.4–38.1)
Place of delivery		
Other (respondents home, other home)	27.6 (1,401)	(26.4–28.9)
Government hospital	19.5 (989)	(18.4–20.6)
Government health center/post	47.6 (2,415)	(46.2–49.0)
Other public sector	0.1 (6)	(0.05–0.3)
Private hospital/clinic	1.2 (60)	(0.9–1.5)
Mission hospital/clinic	4.0 (201)	(3.5–4.5)
Attendance during delivery		
Skilled	68.8 (3,493)	(29.9–37.5)
Not skilled	31.2 (1,581)	(67.5–70.1)
Birth order		
1	21.9 (1,114)	(20.8–23.1)
2	18.0 (908)	(16.7–20.0)
3+	60.1 (3,052)	(58.8–61.5)
Told about pregnancy complications		
Yes	88.1 (4,401)	(87.2–88.9)
No	11.9 (596)	(10.9–12.6)
Wealth status		
Poor	24.6 (1,247)	(29.4–35.8)
Middle	23.0 (1,169)	(21.9–24.2)
Rich	52.4 (2,658)	(51.0–53.7)
Marital status		
Married	80.4 (4,080)	(79.3–81.5)
Not married	19.6 (994)	(18.5–20.7)
Maternal education		
No education	10.5 (533)	(9.7–11.4)
Primary	54.1 (2,740)	(52.7–55.4)
Secondary	31.6 (1,604)	(30.4–32.9)
Higher	3.8 (191)	(3.3–4.3)
Paternal education		
No education	9.2 (418)	(6.1–7.5)
Primary	40.6 (1,837)	(39.1–42.0)
Secondary	43.5 (1,966)	(42.0–44.9)
Higher	6.7 (302)	(6.0–7.4)
Place of residence		
Rural	66.3 (3,363)	(65.0–67.6)
Urban	33.7 (1,711)	(32.5–35.0)
Distance to health facility		
An issue—yes	44.7 (2,264)	(43.3–46.0)
An issue—no	55.3 (2,807)	(54.0–56.7)

*CI, 95% confidence interval*.

*Design was a cross-sectional survey using two-stage sampling method*.

### Determinants of PNC Utilization

The proportion of women who had PNC in the first 48 h after delivery of the baby was 63% (95% CI 61.9–64.6). For women who utilized PNC in the first 48 h, within the age group 20–24 years a proportion representation of 66% while women 35 years or older only (57%) utilized PNC in the first 48 h and this was statistically significant (*p* < 0.001). From those who delivered in health facilities, nearly 84% of those that had their delivery in government hospitals utilized PNC in the first 48 h and 88% of those who delivered in private hospital/clinic had PNC in the first 48 h. Apart from marital status (*p* = 0.767), all the factors considered in this study were associated with PNC utilization. For women who utilized PNC in the first 48 h, within the age group 15–19 years (65%) while women 35 years or older had the least (57%), and this was statistically significant (*p* < 0.001). More women (69%) utilized PNC in the first 48 h after delivery of the baby among those who had their first antenatal care in the first trimester. For women who had skilled attendance during delivery, 82% had PNC utilization in the first 48 h. A higher proportion (65.5%) of women who had autonomy to make decision on their health matters were able to utilize PNC in the first 48 h. First time mothers who were able to utilizing PNC in the first 48 h represented 72% of this group.

Being told about pregnancy complications during antenatal care was associated with PNC utilization (*p* < 0.001) with 66% of women who told utilizing PNC in the first 48 h. Only 47% of women whose wealth status was categorized as poor were able to utilize PNC in the first 48 h. Most women (89%) with higher education were able to utilize PNC in the first 48 h and those who were working had a higher proportion (72%). Among those who indicated that distance to the health facility was not an issue about 73% of them utilized PNC in the first 48 h. Women in urban areas had a larger proportion (82%) of those who utilized PNC in the first 48 h compared to women in rural areas (54%), and this was statistically significant (*p* < 0.001). The results are shown in Table 2. Women who delivered at the health facilities, government hospital (AOR 7.24 95% CI 4.92–11.84), government health center/post (AOR 7.15 95% CI 4.79–10.66), private hospital/clinic (AOR 10.08 95% CI 3.35–30.35) were far more likely to utilize PNC in the first 48 h than those who did not deliver at the health facility. Being attended to by skilled personnel during delivery predicted PNC utilization in the first 48 h. Women who had skilled attendance were twice more likely (AOR 2.30, 95% CI 1.57–3.37) to utilize PNC in the first 48 h. Women who were told about pregnancy complications during their visit at antenatal clinic were more (AOR 1.37, 95% CI 1.04–1.85) likely to utilize PNC in the first 48 h than those were not told. Rural women were less likely (AOR 0.70 95% CI 0.53–0.92) to utilize PNC than women in urban areas. Starting antenatal care in the second or third trimester made it less likely (AOR 0.75, 95% CI 0.53–0.93) for women to utilize PNC compared to those who had their first antenatal care in the first trimester. Wealth status was associated with PNC utilization. Women who were in the middle wealth status (AOR 1.45, 95% CI 0.53–0.92) and rich wealth status (AOR 1.75, 95% CI 0.53–0.92) were more likely to utilize PNC services compared to those who were in the poor category. Table 3 presents the results.

**Table 2:** Factors associated with maternal postnatal care (PNC) utilization in Zambia—results of Pearson’s chi-squared test.

	PNC within first 48 h	PNC after first 48 h	
Characteristic	% (*n*)	% (*n*)	*p*-Value
Age (in years, grouped)			<0.001
15–19	65.2 (440)	34.8 (235)	
20–24	66.3 (866)	33.7 (440)	
25–29	65.3 (820)	34.7 (437)	
30–34	62.1 (581)	37.9 (356)	
35–49	57.0 (512)	43.0 (387)	
Timing of ANC			0.009
First trimester	68.9 (828)	31.1 (373)	
Second and third trimester	63.5 (2,216)	36.5 (1,273)	
Place of delivery			<0.001
Other (respondents home, other home)	17.6 (246)	82.4 (1,155)	
Government hospital	83.5 (826)	16.5 (163)	
Government health center/post	79.7 (1,924)	20.3 (491)	
Other public sector	86.9 (5)	13.1 (1)	
Private hospital/clinic	88.4 (53)	11.6 (7)	
Mission hospital/clinic	81.3 (163)	18.7 (38)	
Birth attendance during delivery			<0.001
Skilled	81.9 (2,860)	18.1 (633)	
Not skilled	22.8 (360)	77.2 (1,222)	
Maternal autonomy for health decision			0.035
Herself	66.5 (844)	33.5 (426)	
Other	61.7 (1,757)	38.3 (1.089)	
Birth order			<0.001
1	71.9 (801)	28.1 (313)	
2	68.0 (617)	32.0 (271)	
3+	59.0 (1,802)	41.0 (1,250)	
Being told about pregnancy complications			<0.001
Yes	66.2 (2,915)	33.8 (1,486)	
No	49.1 (287)	50.9 (298)	
Wealth status			<0.001
Poor	47.2 (589)	52.8 (658)	
Middle	55.7 (651)	44.3 (519)	
Rich	64.5 (663)	35.5 (366)	
Marital status			0.767
Married	63.2 (2,580)	36.8 (1,500)	
Not married	64.4 (640)	35.6 (354)	
Maternal education			<0.001
No education	48.4 (258)	51.6 (275)	
Primary	59.2 (1,621)	40.8 (1,119)	
Secondary	73.0 (1,171)	27.0 (433)	
Higher	88.6 (169)	11.4 (22)	
Maternal occupation			0.005
Not working	66.6 (1,498)	33.4 (753)	
Other	71.7 (721)	28.3 (284)	
Paternal education			<0.001
No education	52.8 (162)	47.2 (145)	
Primary	55.1 (1,013)	44.9 (824)	
Secondary	68.3 (1,342)	31.7 (624)	
Higher	84.6 (256)	15.4 (47)	
Place of residence			<0.001
Urban	81.5 (1,395)	18.5 (316)	
Rural	54.3 (1,825)	45.7 (1,538)	
Distance to health facility			<0.001
An issue––Yes	52.0 (1,177)	48.0 (1,087)	
An issue—No	72.7 (2,040)	27.3 (767)	

*Statistical significance at p < 0.05*.

*CI confidence interval*.

**Table 3:** Key predictors of maternal postnatal care utilization in Zambia—Results of multivariate logistic regression.

Characteristic	OR	95% CI	*p-*Value	AOR	95% CI	*p-*Value
Age (in years, grouped)						
15–19	1					
20–24	1.05	(0.84–1.32)	0.67			
25–29	1	(0.80–1.25)	0.98			
30–34	0.87	(0.69–1.10)	0.25			
35–39	0.71	(0.55–0.91)	**0.007**			
Timing of ANC						
First trimester	1			1		
Second and third trimester	0.76	(0.63–0.91)	**<0.003**	0.75	(0.61–0.93)	**0.007**
Do not know	0.66	(0.21–2.13)	0.489	0.55	(0.13–2.25)	0.399
Place of delivery						
Other (respondents home, other home)	1			1		
Government hospital	23.78	(18.30–30.90)	**<0.001**	7.24	(4.62–11.34)	**<0.001**
Government health center/post	18.38	(14.73–22.92)	**<0.001**	7.15	(4.79–10.66)	**<0.001**
Other public sector	31.13	(5.81–166.97)	**<0.001**	23.2	(3.69–145.91)	**0.001**
Private hospital/clinic	35.75	(12.67–100.86)	**<0.001**	10.08	(3.35–30.35)	**<0.001**
Mission hospital/clinic	20.32	(12.77–32.35)	**<0.001**	8.56	(4.71–15.53)	**<0.001**
Birth attendance during delivery						
Not skilled	1			1		
Skilled	15.33	(12.67–18.55)	**<0.001**	2.30	(1.56–3.40)	**<0.001**
Maternal for health decision						
Herself	1					
Other	0.81	(0.67–0.98)	**<0.031**			
Birth order						
1	1					
2	0.82	(0.66–1.04)	0.11			
3+	0.13	(0.47–0.68)	**<0.001**			
Being told about pregnancy complications						
No	1			1		
Yes	2.02	(1.62–2.52)	**<0.001**	1.39	(1.04–1.85)	**0.028**
Wealth status						
Poor	1			1		
Middle	1.4	(1.17–1.68)	**<0.001**	1.25	(1.00–1.56)	**0.049**
Rich	3.26	(2.73–3.91)	**<0.001**	1.47	(1.17–1.83)	**<0.001**
Marital status						
Married	1					
Not married	1.05	(0.88–1.26)	0.589			
Maternal education						
No education	1					
Primary	1.54	(1.24–1,93)	**<0.001**			
Secondary	2,88	(2.24–3.71)	**<0.001**			
Higher	8.25	(4.84–14.07)	**<0.001**			
Maternal occupation						
Not working	1					
Other	0.79	(0.68–0.92)	**0.003**			
Paternal education						
No education	1					
Primary	1.21	(0.94–1.55)	<0.140			
Secondary	2.11	(1.63–2.74)	**<0.001**			
Higher	5.4	(3.46–8.43)	**<0.001**			
Place of residence						
Urban	1			1		
Rural	0.27	(0.22–0.33)	**<0.001**	0.70	(0.53–0.93)	**0.012**
Distance to health facility						
An issue—yes	1					
An issue—no	2.46	(2.08–2.91)	**<0.001**			

*OR, odds ratio; AOR, adjusted odds ratio*.

The bold font refers to p values which were significant i.e. <0.05

## Discussion

In this study, we sought to investigate the determinants of maternal PNC utilization in the first 48 h after delivery of a baby among childbearing women in Zambia, place of delivery was the main focus considered as a possible determinant, other factors such as place of residence and skilled attendance during delivery were investigated. It was observed that place of delivery was associated with maternal PNC utilization in the first 48 h, emphasizing the importance of women delivering at health facilities. It was found out that place of residence was associated with maternal PNC utilization in the first 48 h, women in rural areas were less likely to utilize PNC, emphasizing the need to reduce the inequities in service provision regardless of residence. It was also observed that skilled attendance during delivery was a predictor of maternal PNC utilization in the first 48 h, stressing the importance having women deliver in health facilities.

Women who delivered in a health facility were seven times more likely to use PNC in the first 48 h after delivery of the baby than those who did not deliver in the health facility. The positive association of place of delivery with PNC services utilization could be attributed to the fact that women who gave their last birth in a health institution had greater opportunity to get exposed to health education related to PNC services at the time of delivery and thus get access to learn about the types, benefits and availabilities of PNC services during their stay in the health institutions (2). Many factors could explain an observation that a proportion of women did not deliver at health facilities and consequently reduced their chances of maternal PNC use in the first 48 h. Studies (13, 15) found out that the low level of complete PNC compliance may arise from a combination of structural determinants, such as poor access to services, perceived lack of services at such facilities by mothers, perceived lack of importance of seeking PNC services, a shortage of community providers making routine home visits, costs and transportation difficulties, and other cultural, geographic, or financial barriers. It was found out that even among the women who delivered in health facilities approximately 20% did not utilize PNC in the first 48 h after the birth of the baby. It would be important thus, to conduct research to establish factors that lead to a proportion of women not utilizing PNC in the first 48 h after despite delivering in a health facility. Future research needs to explore why such opportunities to provide PNC to mothers who deliver in health facilities and deliveries attended by skilled attendant are missed (13).

It was also established that there was reduced likelihood of PNC utilization by women living in rural areas compared to those in urban areas which could be attributed to many factors including distance from health facilities (16), cultural practices, or myths and beliefs. For instance, there are cultural practices which prevent recently delivered mothers and newborn to be touched by any one or leave the house until the 12th day after delivery (17) and have been associated with non utilization of PNC (18). Additionally, compared with women living in rural areas, women living in urban areas have generally better access to PNC services as well as other advantages of urban life, such as greater exposure to health promotion programs (17). From these studies, it is clear that, unless deliberate measures were formulated and implemented to facilitate PNC utilization in rural areas, and especially delivering in health facilities, women in these areas would continue to have low utilization of this service which consequently would predispose them morbidity and mortality. Thus strategies and programs to specifically address these issues would contribute to the improved maternal PNC utilization. Maternal PNC should, for example, be provided by community health workers who are trained to provide such care during routine home visits has for those who did not deliver at health facilities (13).

Being attended to by some skilled personnel during delivery as other studies have found (11, 13) was significantly associated with maternal PNC utilization. Women who were seen by skilled personnel were twice more likely to utilize PNC in the first 48 h than those not seen by any skilled personnel. This would seem to be obvious because skilled personnel would want to ensure that the woman is checked before being allowed to go home. Subsequently, mothers should receive PNC from skilled attendants at least once within 48 h after birth, before being discharged from the place of delivery, as per the Zambian PNC guidelines (10). It is also important that even the nearly 20% of women who were seen by skilled personnel but did not receive the PNC within the first 48 h after delivery were attended to. Missing the opportunities to provide essential PNC in facility deliveries unnecessarily puts the lives of mothers and infants at risk.

Women who were told about pregnancy complications during their ANC visit were more likely to utilize PNC than their counterparts who were not told. Similarly, studies have found out that knowledge of postpartum obstetric danger signs was also found to be a strong predictor of PNC utilization. In one study, mothers who were knowledgeable about at least one postpartum obstetric danger sign were more likely to utilize PNC services as compared to those who did not spontaneously mentioned any postpartum obstetric danger sign (2). This result is similar with the study conducted in Uganda (19). This could be explained by the fact that awareness of obstetric danger signs is an important factor in motivating women and their families to attend health-care service at the earliest opportunity with the intention of prevention, early detection, and getting management of their obstetric danger signs. This emphasized the need to have comprehensive and focused messages during ANC visit that would influence the maternal PNC utilization. Cross-sectional studies like this one, limit the capacity to establish causal inferences. The information that was used was obtained retrospectively, raising the possibility of recall bias; however, this was addressed to some extent by only asking the women who gave birth within the last 2 years prior to the survey.

## Conclusion

It has been demonstrated in this study that maternal PNC utilization in the first 48 h is below the acceptable levels. Women who deliver in health facilities were more likely to utilize PNC in the first 48 h after the delivery of the baby. Thus, there is an urgent need to promote access to health facility delivery, especially so in rural areas where it was even lower than in urban areas. This could help in the reduction of maternal morbidity and mortality in Zambia.

## Ethics Statement

Ethical approval for the 2013/14 ZDHS was obtained from the Tropical Diseases Research Centre (TDRC) in Ndola, Zambia and the US Centre for Disease Control and Prevention (CDC) Atlanta Research Ethics Review Board. Participation in the survey was based on informed and voluntary consent. The re-analysis of the data reported in this study did not infringe on participants’ privacy and was judged to pose no risk, since these data were already de-identified, approved, and made available for public use. Additionally, clearance was obtained from Excellency in Research Ethics and Science (ERES) Committee that granted approval to conduct this study on the factors associated with maternal postnatal care utilization based on the 2013/14 ZDHS (Ref. no. 2016-June-014).

## Author Contributions

CC participated in the conception of the study, drafted the manuscript, cleaned the dataset, and carried out the statistical analysis. MM participated in the critical review of the manuscript and also in the statistical handling and interpretation. CJ participated in the early drafting of the manuscript, interpretation of statistical analysis results, and critical review of the manuscript. MC participated in the review of statistical analysis results, and general review of the manuscript. All authors read and approved the final manuscript.

## Conflict of Interest Statement

The authors declare that the research was conducted in the absence of any commercial or financial relationships that could be construed as a potential conflict of interest.
